# Vaccination of Silver Sea Bream (*Sparus sarba*) against *Vibrio alginolyticus*: Protective Evaluation of Different Vaccinating Modalities

**DOI:** 10.3390/ijms17010040

**Published:** 2015-12-29

**Authors:** Jun Li, Siyuan Ma, Norman Y. S. Woo

**Affiliations:** 1 School of Biological Sciences, Lake Superior State University, Sault Ste. Marie, MI 49783, USA; 2 Key laboratory of Experimental Marine Biology, Institute of Oceanology, the Chinese Academy of Sciences, Qingdao 266071, China; lijun05@gmail.com; 3 Laboratory for Marine Fisheries Science and Food Production Processes, Qingdao National Laboratory for Marine Science and Technology; Qingdao 266071, China; 4 Department of Biology, The Chinese University of Hong Kong, Shatin, N.T., Hong Kong, China; normanwoo@cuhk.edu.hk

**Keywords:** *Sparus sarba*, *Vibrio alginolyticus*, vibriosis, vaccine, vaccination, administration route

## Abstract

In order to develop more effective immunological strategies to prevent vibriosis of farmed marine fish in Hong Kong and southern China, various vaccine preparations including formalin-, phenol-, chloroform- and heat-killed whole cell bacterins and subcellular lipopolysaccharides (LPS), as well as different administration routes, were investigated. Fish immunized with the subcellular LPS exhibited the best protection [Relative Percent of Survival (RPS) = 100], while fish immunized with whole cell bacterins displayed varying degrees of protection (RPS ranged from 28 to 80), in descending order: formalin-killed > phenol-killed > heat-killed > chloroform-killed bacterins. Regarding various administration routes, fish immunized with two intraperitoneal (i.p.) injections exhibited the best protection, and the RPS values were 100 or 85 upon higher or lower doses of pathogenic *V. alginolyticus* challenges. Both oral vaccination and a combination of injection/immersion trial were also effective, which achieved relatively high protection (the RPS values ranged from 45 to 64.3). However, two hyperosmotic immersions could not confer satisfactory protection, especially when fish were exposed to the severe pathogenic bacteria challenge. Marked elevations of serum agglutinating antibody titer were detected in all immunized fish. Macrophage phagocytosis was enhanced significantly, especially in the fish immunized by formalin- and phenol-killed bacterins through various administration routes. Both adaptive (specific antibody) and innate (phagocytic activity) immunity elicited by different immunization strategies were in parallel with the degree of protection offered by each of them. Although all vaccination trials had no significant effect on the serum hematocrit and hemoglobin levels, the circulating lymphocyte counts were significantly elevated in the fish immunized with LPS, formalin- and phenol-killed bacterins. Serum cortisol levels appeared to be reduced in all immunized fish except the trial of hyperosmotic immersion, which indicated the stressful impact on vaccinated fish.

## 1. Introduction

Vibriosis is one of the most important bacterial diseases affecting both marine and freshwater fish species under intensive culture conditions [1,2]. Effective vaccines have been successfully applied in various farmed fish to prevent vibriosis outbreaks caused by a variety of *Vibrio* spp. [3]. In most cases, these vaccines are effective upon administration via oral [4,5,6,7], direct or hyperosmotic immersion [8,9], and injection [4,10]. Other vaccine deliveries such as spray [4], dip or bath [11,12,13,14], or anal intubation [15] have also been reported elsewhere. Protection against vibriosis is accomplished via the stimulation of specific immune responses such as antibody titer [16,17,18,19] and antibody-forming cells [20]. In addition, augmentation of non-specific immune responses such as increased phagocytosis and oxidative radical production in phagocytes, as well as serum lysozyme activity, has also been reported [7,10,21,22]. Vaccination also influences trafficking of immune cells between hemopoietic organs and blood and metabolic parameters such as hematocrit and serum protein levels [10]. The extent of immune responsiveness greatly depends upon bacterial species [19], dose of vaccine [22], and methods of vaccine preparation and administration [14,20,23].

Silver sea bream (*S. sarba*) is a commonly cultured marine fish species in the coastal area of Hong Kong and southeast China. In recent years, however, outbreaks of vibriosis caused by *V. alginolyticus* have become the major cause of losses in the intensive aquaculture system [24,25]. Chemotherapy with antibiotics or other veterinary drugs is far from satisfactory because of the appearance of antibiotic-resistant strains [26]. However, there is as yet no specific vaccine available against *V. alginolyticus*, and commercial vaccine products (against other *Vibrio* spp.) appear to not be effective for controlling vibriosis in local fish farms. Therefore, the main objectives of the present study were: (1) to develop and compare the efficacies of various vaccine preparations against vibriosis in silver sea bream; (2) to evaluate the efficacy of different administration routes; and (3) to determine the potential protective mechanisms induced by each vaccination protocol.

## 2. Results

### 2.1. Safety Assessment

All the vaccine preparations were assessed for safety by i.p. injections in silver sea bream. All the injected fish survived and no abnormal behavior was observed during the two-week period post vaccine injection.

### 2.2. Protection against Pathogenic Challenge

In strategy 1, silver sea bream immunized with whole cell bacterins exhibited varying degrees of protection against pathogenic *V. alginolyticus* challenges (Table 1). Fish immunized with FKC and PKC exhibited better survival than those immunized with CKC or HKC. Fish immunized with LPS extracted from the bacterial cell wall exhibited up to 100% survival. Compared to the control group, the RPS provided by various vaccines was in descending order: LPS > FKC > PKC > HKC > CKC (Table 1).

**Table 1:** Comparative efficacy of different vaccines against *Vibrio alginolyticus* in silver sea bream, *Sparus sarba*.

Vaccines	Number of Vaccinated Fish	Number of Challenged Fish	Number of Dead Fish	Mortality (%)	RPS
*Whole Cell Bacterins*					
Formalin-killed	36	35	7	20	80
Phenol-killed	36	34	8	23.5	76.5
Chloroform-killed	36	35	25	71.4	28.6
Heat-killed	36	36	18	50	50
*Subcellular Bacterins*					
LPS	36	36	0	0	100
Control	36	36	36	100	0

LPS = lipopolysaccharide; RPS = relative percentage of survival.

**Figure 1:**
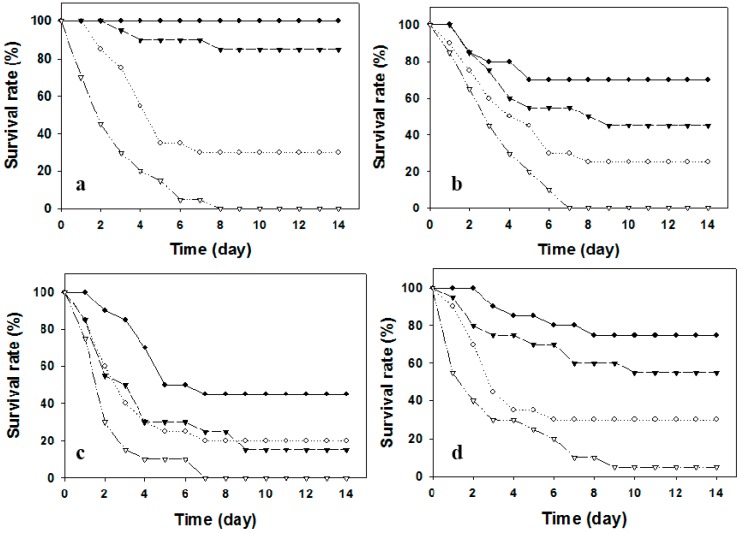
The kinetics of survival rates of silver sea bream (*N* = 30–33) against virulent *Vibrio alginolyticus* challenges after immunization with formalin-killed bacterins through (**a**) i.p. + i.p.; (**b**) i.p. + imm.; (**c**) imm. + imm.; and (**d**) oral administration routes. (●―●, vaccinated fish, and ○⋯○, control, both groups were challenged i.p. with the bacterial dose of 5.0 × 10^5^ cfu/fish; ▼--▼, vaccinated fish, and ∇-··-∇, control, both groups were challenged i.p. with the bacterial dose of 5.0 × 10^7^ cfu/fish).

**Figure 2:**
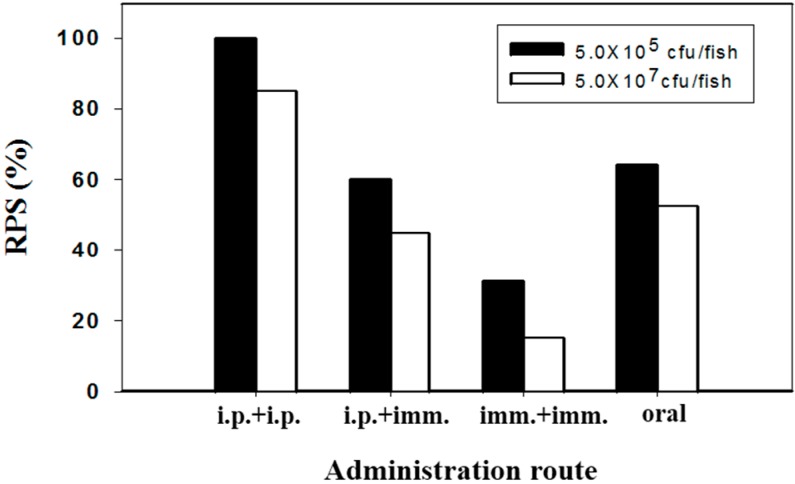
Protection (RPS) of silver sea bream (*N* = 30–33) against virulent *V. alginolyticus* challenge after three weeks of vaccination with formalin-killed bacterins through different administration routes. (RPS = (1 − mortality of vaccinated fish/mortality of control fish) × 100).

In respect to various delivery routes, both vaccinated and related control fish in strategy 2 were injected intraperitoneally with two different doses of pathogenic *V. alginolyticus* (strain Vib-01). The kinetic survival rates were shown in Figure 1. The pathogenic bacteria of Vib-01 were reisolated and confirmed from moribund or/and dead fish as previously described [25]. Almost all (over 95%) unvaccinated fish (control groups) died upon the bacterial challenge at a dose of 5 × 10^7^ cfu/fish, whereas 20%–30% of fish survived with a lower dose (5 × 10^5^ cfu/fish) bacterial challenge (Figure 1). However, immunized fish exhibited varying degrees of protection against pathogenic *V. alginolyticus*. In the case of higher challenge doses, fish immunized by two immersions exhibited an unsatisfactory survival (15%), while better protections were achieved in the trials of fish immunized orally (survival = 55%) or under the “i.p. + imm.” regime (survival = 45%). The best survival rate (85%) was conferred by two injections of FKC. However, once challenged with a lower bacterial dose (5 × 10^5^ cfu/fish), significantly higher survival rates were observed in all immunized fish (Figure 1). Irrespective of the challenge dose, the RPS offered by different vaccination trials was in the descending order of “i.p. + i.p.” > o.v. > “i.p. + imm.” > “imm. + imm.” (Figure 2).

### 2.3. Specific Antibody Titer

**Figure 3:**
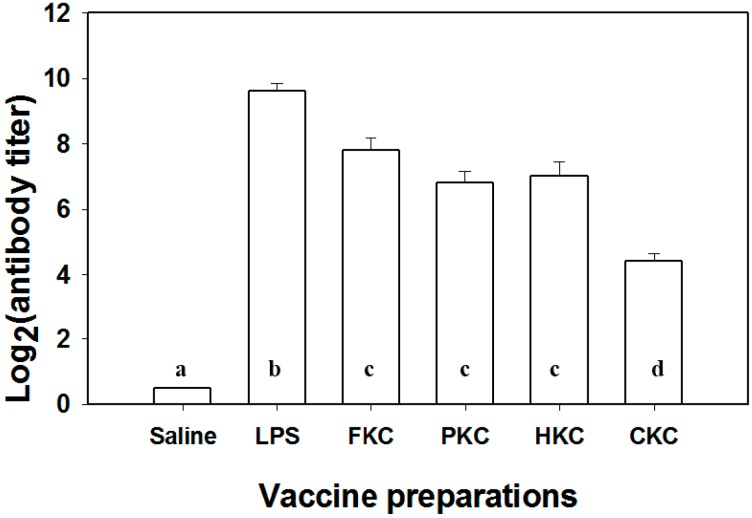
Agglutinating antibody titer of the sera from *Sparus sarba* immunized i.p. with various vaccines. Groups with different alphabets are significantly different (*p* < 0.001) from each other. LPS: lipopolysaccharides; FKC: formalin killed cells; PKC: phenol killed cells; HKC: heat killed cells; CKC: chloroform killed cells.

**Figure 4:**
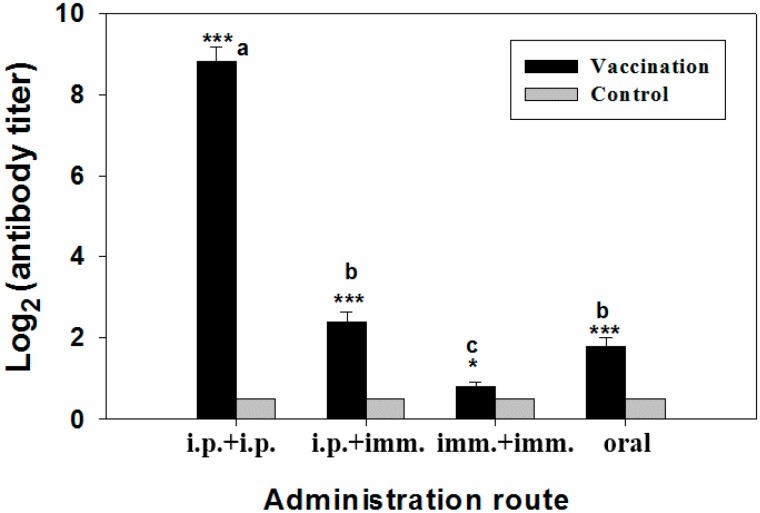
Serum agglutinating antibody titers in silver sea bream (*N* = 7) immunized with formalin-killed bacterins through different administration routes. Significant differences from control were expressed as * *p* < 0.05 and *** *p* < 0.001. Significant differences among groups of immunized fish were shown as a, b, and c (a > b > c).

The specific immune response in silver sea bream after vaccination was evaluated by measuring serum agglutinating antibody titer. In strategy 1, all the immunized fish exhibited significantly higher (*p* < 0.001) agglutinating antibody titers in comparison with the control (Figure 3). Fish immunized with LPS displayed the highest antibody level, whereas fish immunized with CKC bacterins exhibited the lowest antibody level among all the immunized groups. Intermediate antibody levels were induced by the other three whole cell bacterins (FKC, PKC and HKC) with no significant difference among them (Figure 3). As in strategy 2, serum antibody titers were almost undetectable (<2) in unvaccinated (saline-injected) fish (Figure 4). However, the values of serum agglutinating antibody were significantly elevated in all immunized fish in comparison to controls (Figure 4). Fish immunized twice with i.p. injections exhibited the highest level of agglutinating antibody, while fish vaccinated by both immersions produced the lowest antibody titers. Moderate antibody titers were achieved in the fish immunized orally or by “i.p. + imm.”; however, no significant difference was found between the two trials (Figure 4).

### 2.4. Macrophage Phagoctic Activity

**Figure 5:**
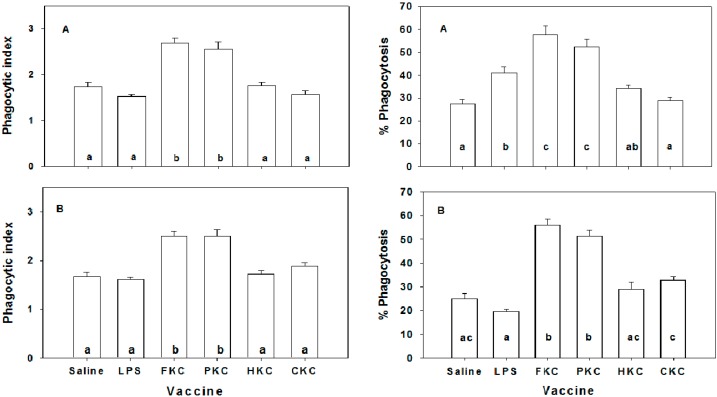
Phagocytic indices and percent of phagocytosis of pronephic (**A**) and splenic (**B**) macrophages of *Sparus sarba* immunized i.p. with various vaccines. Groups with different alphabets are significantly different (*p* < 0.05) from each other. LPS: lipopolysaccharides; FKC: formalin killed cells; PKC: phenol killed cells; HKC: heat killed cells; CKC: chloroform killed cells.

**Figure 6:**
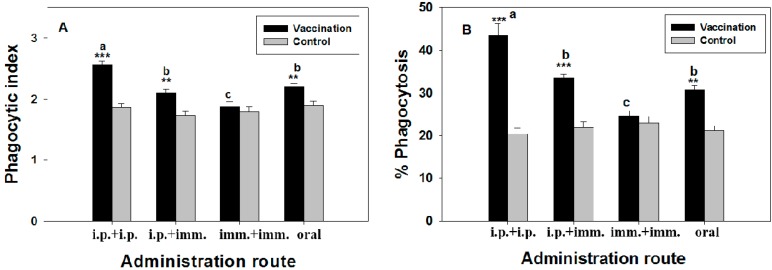
Phagocytic activities ((**A**) phagocytic index, (**B**) percent phagocytosis) of pronephric macrophages of silver sea bream (*N* = 7) immunized with formalin-killed bacterins via different administration routes. Significant differences from control were expressed as ** *p* < 0.01 and *** *p* < 0.001. Significant differences among groups of immunized fish were shown as a, b, and c (a > b > c).

The non-specific immune response of silver sea bream after vaccination was determined by assessing macrophage phagocytic activity. In strategy 1, inoculation of FKC and PKC bacterins caused significant increases in phagocytic indices in both splenic and pronephric macrophages (Figure 5). However, there were no significant effects of the other vaccines on the macrophage phagocytic indices by comparing them to controls (Figure 5). Similarly, macrophage phagocytosis was significantly elevated in the fish immunized with FKC and PKC bacterins. Apart from a significant increase in the percent of phagocytosis of pronephric macrophages taken from fish immunized with LPS, all changes in the percent of phagocytosis of both splenic and pronephric macrophages induced by LPS-, HKC- and CKC- inoculations were not significantly different. Similarly, in strategy 2, macrophage phagocytic activities were markedly enhanced in silver sea bream immunized by “i.p. + i.p.”, “i.p. + imm.” and oral vaccination routes (Figure 6). However, the increments in macrophage phagocytosis in fish immunized by the trial of double immersions were not statistically different from those of control fish (Figure 6). Among different vaccination trials, fish immunized by two injections of vaccine exhibited the highest macrophage phagocytic activities. Both phagocytic index and the percent of phagocytosis in the fish immunized by the routes of o.v. and “i.p. + imm.” were similar; however, the values in both groups were significantly higher than those of “imm. + imm.” vaccinated fish (*p* < 0.05). Macrophage phagocytosis in all unvaccinated groups remained constant at the basal level (oral-control) (Figure 6).

### 2.5. Hematology and Organ Weight

Fish inoculated with different vaccines exhibited a variety of effects on the spleno-somatic indices (Supplementary Table S1). When compared with the respective controls, the indices of SSI were significantly reduced by 52% and 45.8%, respectively, in fish immunized with FKC and PKC bacterins. There was a general trend of elevated SSI values in fish immunized with HKC and CKC bacterins and LPS, but the changes were only significantly higher in CKC-injected fish when compared with the saline-injected controls (Supplementary Table S1).

Immunization with various vaccines resulted in no significant changes in both hematocrit and hemoglobin values in silver sea bream with the exception of a significant increase in hemoglobin occurring in FKC-inoculated fish. The circulating lymphocyte counts were significantly higher (*p* < 0.001) in those fish vaccinated by LPS, FKC and PKC in comparison with saline-injected controls, while no significant changes occurred in the fish immunized with CKC and HKC bacterins (Supplementary Table S1).

**Figure 7:**
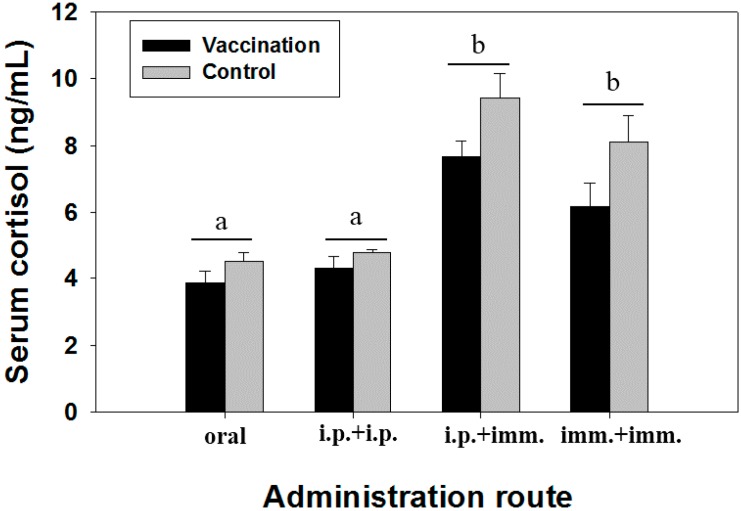
Serum cortisol of silver sea bream (*N* = 7) immunized with formalin-killed bacterins through different administration routes. Significant difference among groups of vaccinated fish or unvaccinated fish was shown as a & b (b > a).

Irrespective of the route of vaccine administration, the number of red blood cells remained unchanged in all immunized fish when compared to control groups (Supplementary Table S2). The total leukocyte counts were significantly elevated in fish immunized with two injections or with the first injection followed by immersion vaccination. However, the changes of the white blood cells were not significant in the fish immunized by double immersions or by oral administration (Supplementary Table S2). Values of hematocrit and hemoglobin did not alter significantly in the fish with or without vaccination (Supplementary Table S2). Except for a slight decrease of serum cortisol in vaccinated fish, no significant impacts of immunization were observed between vaccinated and unvaccinated fish (Figure 7). However, cortisol levels in the fish treated by “i.p. + imm.” or “imm. + imm.” trials, irrespective of receiving the vaccine or not, were significantly (*p* < 0.05) stimulated when compared with those induced by oral or double-injection treatments (Figure 7).

HSI and RSI exhibited a general trend of elevation in vaccinated fish; however, the increases in HSI in fish that received “i.p. + imm.” vaccination and RSI in orally vaccinated fish were not statistically significant (Supplementary Table S2). Variation in SSI was observed in the fish immunized by different administration routes. i.p. injections of vaccine elicited a 3.36-fold elevation in SSI over that of control fish. Although oral vaccination also could induce a slight increase of SSI, the alteration was not statistically significant. In contrast, other vaccination methods such as “i.p. + imm.” and “imm. + imm.” suppressed SSI in silver sea bream; however, the declinations were not significant when compared to controls (Supplementary Table S2).

## 3. Discussion

Vaccination has become a routine prophylactic treatment for preventing various fish diseases in intensive mariculture [11,22,27,28]. In the present study, we have evaluated the protective efficacies and the potential immune mechanisms of five different vaccine preparations as well as different administration routes in silver sea bream against *V. alginolyticus*. All the candidate vaccines appeared safe to silver sea bream and the immunized fish exhibited varying degrees of protection after experimental challenge with virulent *V. alginolyticus*. 

Under the i.p. delivery route, fish immunized with LPS acquired the best protection, whereas the other vaccine preparations gave variable protective effects upon pathogenic *V. alginolytics* challenges. Varied efficacy offered by different vaccine preparations have been reported in a variety of fish against vibriosis [3]. The highest level of specific antibody titer and circulating lymphocyte count in LPS-vaccinated fish may explain the highest efficacy of protection among various vaccine preparations. Enhancement of macrophage phagocytosis has also been considered to contribute to the protective immunity of fish immunized with LPS [21,28,29,30], but no significant change in macrophage phagocytosis was observed in silver sea bream vaccinated with LPS [31]. Our results suggest that the highest protection against *V. alginolyticus* offered by LPS was mainly due to the induction of specific humoral immunity in silver sea bream. 

Formalin- or heat-killed whole cell bacteria are currently the most popular vaccines employed in farmed fish, and good protection against vibriosis has been achieved in a variety of fish species worldwide [3], including silver sea bream immunized with formalin-killed *V. alginolyticus* in our present study. Relevant higher values of agglutinating antibody titer, lymphocyte count and macrophage phagocytic activity were found in the fish immunized with FKC, a finding which was similar to previous reports of other fish species [6,21,32,33,34]. However, fish immunized with CKC bacterins evoked very weak protection, and the levels of induced antibodies and the lymphocyte count, as well as macrophage phagocytosis, were very low. These findings are in contrast to previous reports [35,36] in which CKC was effective in protecting the immunized fish against pasteurellosis and enteric redmouth disease (ERM). Moreover, our finding that higher protection (RPS = 76.5) and significant elevation in both specific and non-specific immune responses produced by PKC suggested that PKC was a good candidate vaccine for silver sea bream against *V. alginolyticus*. It can therefore be concluded that, in addition to the humoral response, other mechanisms, such as the non-specific immune responses, seem to play important roles for silver sea bream in response to the whole cell bacterins. 

Available evidence has shown that the major immunogens of whole cell bacterins are lipopolysaccharides (LPS), an essential component of the outer membrane of Gram-negative bacteria [22,37,38,39]; however, it has been demonstrated that the dominant immunogens of *V. salmonicida* are the surface layer, a protein-LPS complex (VS-P1) [40,41]. The lipopolysaccharides are large, heat-stable molecules that are resistant to the harsh conditions used for their extraction and purification. However, the potential antigenic proteins (low molecular weight, e.g., 40 kDa VS-P1) are perhaps heat-labile and unable to withstand severe extraction methods. This mechanism may explain the varying degrees of protective immune responses of different vaccines in our study due to the different properties by virtue of different methods of preparation. More experiments are required for delineating the protective mechanisms of whole cell and subcellular vaccines in silver sea bream against *V. alginolyticus*. 

Generally, the extent of immune responsiveness induced by vaccination depends on the physiological status of the fish, the bacterial species from which the vaccines are prepared, the methods of vaccine preparation, and the dose and route of vaccine administration [4]. In our present study, fish vaccinated with FKC via different delivery routes have also resulted in varying degrees of protection (RPS values ranged from 15 to 100) against pathogenic *V. alginolyticus* challenges. Similar to the previous findings that i.p. injection was the most common and effective means for fish vaccination [3,4,7,20,42], two i.p. injections offered the best protection in the present study against the pathogenic *V. alginolyticus* challenge. In the case of hyperosmotic immersion, the immunized fish exhibited partial protection against low-dose but inefficient to high-dose pathogenic *V. alginolyticus* challenge. The varying degrees of protection offered by immersion vaccination have been reported previously [20,43,44]. However, the combination of injection plus a booster immersion exhibited a relatively high level of protection. Similar results have been reported in Atlantic salmon (*Salmo salar*) [43] and in turbot (*Scophthalmus maximus*) [17]. All these findings suggest that a single injection seems likely to be necessary for achieving effective protection against vibriosis. 

Although oral vaccination could not offer protection as good as that of injection [3,4,5,6], oral vaccination appeared effective for protecting silver sea bream against pathogenic *V. alginolyticus*. These findings suggest that oral vaccination would be a very promising routine in protecting fish against vibriosis, especially in farming conditions, where the disease is seldom as virulent as the experimental challenge [5,7].

To elucidate the protective mechanisms, silver sea bream immunized by two i.p. injections resulted in a marked increase in serum agglutinating antibody titer and macrophage phagocytic activity. Mounting a specific antibody response and macrophage phagocytic activity against a pathogen is normally an important step in surviving the disease [45]; therefore, the highest levels of both agglutinating antibody titer and macrophage phagocytic activity observed in our present study may contribute significantly to the highest protection against pathogenic *V. alginolyticus* challenge.

Interestingly, we have observed in our study that stimulated macrophage phagocytic activity occurred as early as 12 h after a single i.p. vaccination and, at that time, the agglutination antibody titer was still low or undetectable. Thus, the early activated macrophage prior to the elevation of specific serum antibody may play an essential role at the first line of immunological defense against *V. alginolyticus* in the sea bream.

In the case of the immersion vaccination, the “imm. + imm.” regime could not provide a remarkable elevation in the agglutinating antibody titer and macrophage phagocytosis in silver sea bream. The findings may be responsible for producing the lowest protection in the immunized fish in our study. In addition, significant elevation of the serum cortisol conferred by the hyperosmotic treatment might cause immunosuppressive effects on the immunized silver sea bream [46].

So far, the protective mechanisms involved in the oral vaccination of fish are poorly understood. Since specific antibodies in orally vaccinated fish are very low or undetectable, the non-specific humoral or cellular factors should play more important roles in this type of immune protection [5,7,47]. However, significant increments in both the agglutinating antibody titer and the macrophage phagocytic activity were induced in silver sea bream after oral immunization, which seems to be responsible for the relatively high degree of protection attained in our study. Previous studies suggest that degradation of the vaccine in the digestive tract occurred before uptake by enterocytes and, hence, lower amounts of antigen reaching the immune system of fish might be the main reason for the lower efficacy of oral vaccination [48,49,50]. In the present study, a large dose (5 mg dried bacteria/g body weight/day) of bacterins has been used in oral vaccination; thus, even after degradation in the digestive tract, there will still be enough antigens to reach the hindgut [48,50]. In addition, the stimulation of mucosal immunity has been recently recognized for its essential role in protecting fish against infectious diseases [51,52,53]. However, the details of antigen uptake and the extent of the involvement of mucosal immunity in offering protection are still unclear in silver sea bream immunized by oral and immersion trials.

In silver sea bream, enhanced phagocytic activity and agglutinating antibody titer were associated with elevated HSI and RSI after vaccination, while changes in SSI caused by different methods of vaccination were variable. Further studies are needed to explain what induces the fluctuation in SSI and the correlation between the changes of different organ mass and fish immune response upon different vaccinations. In addition, the significant elevation of leukocyte counts only in response to injectable bacterins in silver sea bream suggests that more antigens had been taken up by the injected fish.

## 4. Materials and Methods

### 4.1. Experimental Fish

Silver sea bream juveniles, weighing approximately 50–100 g, were obtained from a local fish farm in Hong Kong. Fish were transported to the laboratory and acclimated in seawater (33 ppt) aquaria (200,000 L capacity) equipped with biological filtration for three weeks prior to experiments. Fish, without any apparent infectious symptoms, were then randomly separated into experimental groups and maintained in tanks (500 L capacity) equipped with seawater recirculation. Fish were fed *ad libitum* with a pellet diet formulated in the laboratory at the ration size of 5% (fish body weight/day) [54]. Throughout the experimental period, seawater temperature was kept at 20–22 °C and salinity was maintained constant at 33 ppt.

### 4.2. Bacterial Cultures 

The pathogenic *V. alginolyticus* (strain Vib-01) in silver sea bream has been isolated, characterized and cultured in our laboratory [24,25]. Stock cultures were preserved at −80 °C in 15% (*v*/*v*) glycerol-tryptic soy broth (TSB, Difco Laboratories, Detroit, MI, USA). The bacteria were re-inoculated on 2216 E marine agar (Difco Laboratories, Detroit, MI, USA) prior to use. Working cultures of *V. alginolyticus* were obtained by inoculating bacteria in 2216 E broth (5 g peptone, 1 g yeast extract, 0.1 g Fe_3_PO_4_ per liter of aged seawater, pH 7.8) and incubated with shaking at 28 °C for 48 h. The viable counts of the *V. alginolyticus* cultures were estimated by a standard plate count method as described previously [24]. The bacterial culture was washed three times with sterile saline (0.85% NaCl) and then resuspended in saline at the final concentration of 10^9^ cfu/mL.

### 4.3. Vaccine Preparation

Four kinds of whole cell bacterins, which were designated as formalin-killed bacterins (FKC), chloroform-killed bacterins (CKC), phenol-killed bacterins (PKC) and heat-killed bacterins (HKC), were respectively prepared by treating bacterial suspensions (10^9^ cfu/mL) with 0.5% formalin overnight, 1.5% chloroform for 6 h, 1.0% phenol for 2 h at 28 °C, or heating at 65 °C for 3 h. The bacterial suspensions were then washed three times with sterile saline (0.85%, NaCl) by centrifugation at 12,000× *g* for 10 min. The pellets were resuspended in equal volumes of sterile saline and maintained at 4 °C until used in fish vaccination studies. The inactivated bacteria suspension was used as injection and immersion vaccine. To prepare the oral vaccine, bacterial pellets were lyophilized and mixed with fish meal (1:10 *w*/*w*). The mixture was then used as the main ingredient for preparing a moist pellet feed for sea bream [54]. These vaccine preparations were maintained at 4 °C until used.

The subcellular vaccine of lipopolysaccharides (LPS) was prepared using the water-phenol extraction method [55]. Briefly, a working bacterial culture of *V. alginolyticus* was harvested and resuspended in 300 mL distilled water. The bacteria suspension was heated at 65 °C and mixed with an equal volume of pre-heated soluble phenol (90%) with vigorous shaking for 30 min. The mixture was cooled on ice. After centrifugation at 3000 rpm for 30 min, the water phase was collected and the phenol phase which included insoluble residues was mixed again by adding distilled water. This procedure was repeated two times. All the aqueous fractions were pooled together and dialyzed for 48 h against distilled water to remove phenol and small amounts of low molecular weight materials. The sample was then lyophilized and resuspended in distilled water to a final concentration of 5 mg/mL and stored in aliquots at −20 °C. This crude LPS preparation was used directly for fish vaccination.

The safety of various vaccine preparations was assessed in silver sea bream prior to vaccination trials. Groups of fish (10 fish per group) were injected intraperitoneally (i.p.) with FKC, PKC, CKC and HKC whole cell bacterins at 0.2 mL/fish (10^9^ cfu/mL) and LPS at the dose of 1 mg/100 g body weight (0.2 mL/fish). All the fish were maintained under the same conditions as described and monitored for 14 days to ensure the absence of any toxic effects to fish.

### 4.4. Immunization Strategies

**Strategy 1:** Two sets of fish were set up for vaccination. Each set includes six groups, and there were 36 fish per group in the first set, and seven fish per group in the second set. Both sets of fish received the same vaccination protocol. Four groups of fish in each set were vaccinated intraperitoneally (i.p.) with FKC, PKC, CKC and HKC whole cell bacterins at 0.2 mL/fish (10^9^ cfu/mL), respectively. The fifth group of fish was immunized i.p. with LPS at a dose of 1 mg/100 g body weight. The final group of fish was injected i.p. with 0.2 mL/fish sterile saline (0.85% NaCl) as control. One week later, booster vaccination was performed in the same way. Three weeks post-booster vaccination, the first set of fish was applied for pathogenic challenge and the second set was used for immunological analysis.

**Strategy 2:** Groups of fish (50 fish per group) were immunized with formalin-killed whole cell bacterins (FKC) through different administration routes, such as injection, hyperosmotic immersion, injection/immersion combination and oral vaccination (o.v.). The first experimental trial was designated as “i.p. + i.p.”. Fish were injected intraperitoneally (i.p.) with FKC at a dose of 0.2 mL/fish (10^9^ cells/mL). Another group of fish which received 0.2 mL/fish of sterile saline (0.85%, NaCl) served as control. One week later, the vaccinated group received another i.p. injection of booster in the same way as the initial vaccination. The second trial was designated as “imm. + imm.”. Fish were held in a hyperosmotic environment (80 ppt seawater) for 5 min and subsequently vaccinated by immersion in the FKC suspension diluted (1:10) with seawater (33 ppt) for 3 min. Another group of fish was maintained in seawater (33 ppt) to act as control. The vaccinated fish received a similar booster immersion after one week. The third trial was designated as “i.p. + imm.”, in which fish were immunized i.p. firstly with FKC as previously described or with an i.p. injection of sterile saline (control) and followed by immersion in 1:10 diluted FKC suspension or in 33 ppt seawater for control. The fourth group of fish was orally vaccinated and designated as “o.v.”. Here fish were fed with the artificial pellet diet containing vaccine (1:10 *w*/*w* in fish meal) at a ratio of 5% (body weight/day) for seven days, and then the diet was changed to normal fish diet without vaccine. Control fish were fed the normal pellets at the same ratio size. Three weeks after booster vaccination, seven vaccinated fish and seven control fish from each group were removed and sacrificed for hematological and immunological analysis. The remaining fish in each group were separated into two tanks and applied for pathogenic challenge studies.

### 4.5. Pathogenic Challenge

A fresh 24 h bacterial culture of a virulent *V. alginolyticus* (Vib-01) used for experimental challenge was prepared as described elsewhere [25]. All fish were injected intraperitoneally with 0.2 mL/fish of *V. alginolyticus* (Vib-01) at the dose of 5 × 10^5^ or 5 × 10^7^ cfu/fish, respectively. Fish mortality was monitored daily for two weeks. The survival rates and the protection (expressed as relative percent survival (RPS)) of silver sea bream immunized with various vaccine preparations and different administration routes were evaluated according to the following formula described by Amend [56]:

(1) RPS = (1 − mortalities of vaccinated fish/mortalities of control fish) × 100

### 4.6. Hematological Parameters and Organ Weights

Blood samples were taken by caudal vein puncture and immediately transferred into sterile 1.5 mL Eppendorf tubes. A portion of blood was taken into heparinized microhematocrit tubes and spun to obtain the hematocrit value. Meanwhile, an aliquot of blood was immediately diluted with Drabkin’s solution, mixed and stored for 10 min at room temperature. Hemoglobin concentration was then determined by the cyanmethemoglobin method as previously described [25]. Another aliquot of whole blood was immediately diluted and total erythrocyte and leukocyte counts were determined according to standard procedures with a Neubauer hemocytometer. The resting blood was allowed to clot at room temperature for 1 h. Serum was carefully collected after a centrifugation at 10,000× *g* for 10 min. The serum samples were then aliquoted, immediately deep frozen into liquid nitrogen and stored at −85 °C until use.

After blood withdrawal the fish was killed by spinal transection and the liver, pronephros and spleen were excised and weighed. Consequently, the splenosomatic

(2) SSI = (spleen weight ÷ body weight) × 100

renosomatic

(3) RSI = (kidney weight ÷ body weight) × 100

and hepatosomatic

(4) HSI = (liver weight ÷ body weight) × 100

indices were calculated on the basis of spleen, kidney, liver and body weights of fish.

### 4.7. Serum Cortisol

Serum cortisol was assayed using ELISA kits purchased from IBL (Hamburg, Germany) according to manufacturer’s instructions.

### 4.8. Agglutinating Antibody Titer

The agglutinating titers of serum antibody were determined according to the method of Roberson [57]. Briefly, 50 μL serum was serially diluted with 0.85% NaCl in a 96-well microtiter plate with thorough mixing. Then 50 μL of formalin-inactivated *V. alginolyticus* (10^9^ cfu/mL) was mixed into each well and the whole plate was incubated at 28 °C for 2 h and then stored at 4 °C overnight. The agglutination reaction was observed at 20× magnification under a dissecting microscope and the last dilution of serum giving a visible precipitation was taken as the agglutinating antibody titer.

### 4.9. Macrophage Phagocytosis

Macrophage phagocytic activities were assessed according to Deane *et al.* [46]. Briefly, the spleen and pronephros were excised and macerated in Leibovitz-15 (L-15) medium supplemented with 0.1% fetal calf serum (FCS) and 4 U/mL heparin. The resultant cell suspension was incubated on a glass slide for 90 min at 20–22 °C to facilitate the adherence of macrophages to the slide. Non-adherent cells were then washed off using 0.15 M phosphate-buffered saline (PBS) pH 7.2. The preparation was then presented with 2 × 10^8^ heat-killed yeast (*Saccharomyces cerevisiae*) in L-15 medium containing 4% FCS. Phagocytosis was allowed to proceed for 90 min and the preparation was fixed in methanol and stained with May-Grunwald/Giemsa stain. The number of yeast cells engulfed by 200 macrophages were counted and expressed as the phagocytic index and the number of macrophages which engulfed one or more yeast cells represented the percent phagocytosis.

### 4.10. Statistics

All data were expressed as mean values ± S.E.M. and significant difference among groups was estimated using one-way ANOVA followed by the Student-Newman-Keul multiple comparison test (SigmaStat statistical software, Jandel Scientific, San Jose, CA, USA)

## 5. Conclusions

In conclusion, vaccination is an effective treatment to protect farmed silver sea bream against vibriosis caused by pathogenic *V. alginolyticus* by mounting both non-specific and specific immune responses. Effective protections could be offered by two i.p. injections of crude LPS or formalin- or phenol-inactivated whole cell bacterins. Oral vaccination could be considered efficacious in the protection of silver sea bream against vibriosis, particularly in farming conditions where the disease is seldom as virulent as the experimental challenge.

## Supplementary Materials

Supplementary materials can be found at http://www.mdpi.com/1422-0067/17/1/40/s1.
