# Supplementary material for: Vaccination of Silver Sea Bream (Sparus sarba) against Vibrio alginolyticus: Protective Evaluation of Different Vaccinating Modalities
Source: Int J Mol Sci. 2015 Dec 29;17(1):40. doi: 10.3390/ijms17010040 (PMC4730285; doi:10.3390/ijms17010040)
Supplement: Supplementary file 1 [file ijms-17-00040-s001.pdf]

Supplementary Materials: Vaccination of Silver Sea Bream (*Sparus sarba*) against *Vibrio alginolyticus*: Protective Evaluation of Different Vaccinating Modalities

Jun Li, Siyuan Ma and Norman Y. S. Woo

Table S1. Effects of inoculation of different vaccines on the spleno-somatic index (SSI) and hematological parameters in silver sea bream, *Sparus sarba*.

Vaccines	SSI (%)	Hematocrit (%)	Hemoglobin (g/dL)	Lymphocyte (k/mm^3)
Saline	0.048 ± 0.004	46.8 ± 2.4	7.90 ± 0.43	30.4 ± 1.6
LPS	0.040 ± 0.001	37.9 ± 3.5	8.00 ± 0.45	47.9 ± 3.5 ***
Formalin-killed	0.023 ± 0.002 ***	52.2 ± 1.7	10.6 ± 0.6 *	42.9 ± 1.8 ***
Phenol-killed	0.026 ± 0.003 **	49.9 ± 1.8	8.97 ± 0.69	42.4 ± 2.0 ***
Heat-killed	0.059 ± 0.005	37.7 ± 3.0	8.78 ± 0.59	28.7 ± 1.4
Chloroform-killed	0.071 ± 0.004 ***	41.9 ± 1.2	8.93 ± 0.46	30.0 ± 2.1

Significant difference from saline injected group at * $p < 0.05$, ** $p < 0.01$ and *** $p < 0.001$.

Table S2. Hematological parameters and HSI, RSI and SSI values of silver sea bream immunized with formalin-killed bacterins through different administration routes. ($N = 7$).

Immunization Route	HSI (%)	RSI (%)	SSI (%)	Hct (%)	Hb (g/100 mL)	RBC (×10^6 cells/mL)	WBC (×10^3 cells/mL)
i.p. + i.p.-V	0.77 ± 0.12 *	0.38 ± 0.02 *	0.131 ± 0.030 *	31.39 ± 1.10	7.31 ± 0.54	4.26 ± 0.28	11.5 ± 1.23 *
i.p. + i.p.-C	0.49 ± 0.03	0.27 ± 0.01	0.039 ± 0.004	32.88 ± 1.23	6.81 ± 0.37	4.44 ± 0.19	7.00 ± 1.41
i.p. + imm.-V	0.80 ± 0.10	0.27 ± 0.01 *	0.051 ± 0.017	33.42 ± 2.21	9.62 ± 0.62	4.67 ± 0.19	11.2 ± 2.71 **
i.p. + imm.-C	0.69 ± 0.03	0.23 ± 0.007	0.071 ± 0.006	32.51 ± 2.70	8.26 ± 0.36	4.61 ± 0.18	3.00 ± 0.55
imm. + imm.-V	0.75 ± 0.04 *	0.26 ± 0.01 *	0.047 ± 0.008	29.69 ± 2.83	7.99 ± 0.71	4.75 ± 0.28	7.20 ± 2.71
imm. + imm.-C	0.60 ± 0.02	0.22 ± 0.005	0.056 ± 0.011	28.60 ± 1.38	9.32 ± 0.48	4.13 ± 0.14	5.00 ± 0.32
Oral-V	1.07 ± 0.09 *	0.28 ± 0.01	0.054 ± 0.017	27.10 ± 2.26	7.36 ± 0.54	4.17 ± 0.29	6.20 ± 2.08
Oral-C	0.75 ± 0.03	0.24 ± 0.03	0.041 ± 0.011	29.46 ± 1.01	7.90 ± 0.42	4.09 ± 0.14	6.40 ± 1.50

Significant difference from saline injected group at * $p < 0.05$ and ** $p < 0.01$.
